# Metabolic control of ovarian function through the sympathetic nervous system: role of leptin

**DOI:** 10.3389/fendo.2024.1484939

**Published:** 2025-02-03

**Authors:** Camila Astudillo-Guerrero, Alfonso H. Paredes, Jorge Escobar, Daniela Fernandois, Rafael Barra, Gonzalo Cruz

**Affiliations:** ^1^ Laboratorio de Alteraciones Reproductivas y Metabólicas, Instituto de Fisiología, Facultad de Ciencias, Universidad de Valparaíso, Valparaíso, Chile; ^2^ Center for Neurobiochemical Studies in Endocrine Diseases, Laboratory of Neurobiochemistry, Department of Biochemistry and Molecular Biology, Faculty of Chemistry and Pharmaceutical Sciences, Universidad de Chile, Santiago, Chile; ^3^ Laboratorio de Química Biológica, Instituto de Química, Pontificia Universidad Católica de Valparaíso, Valparaíso, Chile; ^4^ Univ. Lille, Inserm, CHU Lille, Laboratory of Development and Plasticity of the Neuroendocrine Brain, Lille Neuroscience & Cognition, UMR-S1172, EGID, DISTALZ, Lille, France; ^5^ Centro de Investigación Biomédica y Aplicada (CIBAP), Escuela de Medicina, Facultad de Ciencias Médicas, Universidad de Santiago de Chile, Santiago, Chile

**Keywords:** sympathetic, ovary, leptin, hypothalamus, metabolic

## Abstract

The link between metabolism and reproduction is well-known. Both undernutrition and obesity affect the reproductive system. Metabolic status influences reproductive physiology by regulating gonadotropin secretion and affecting reproductive organs through hormonal signals. On the other hand, the autonomic nervous system controls follicle development and ovulation in the female reproductive system. This system is regulated by hypothalamic areas associated with metabolism as the Arcuate nuclei (ARC) and paraventricular nuclei (PVN). Metabolic signals, such as nutrients and hormones, acting on the hypothalamus may play a crucial role in modulating sympathetic innervation of the ovary and other reproductive organs. Some of these hormones are leptin, insulin, and GLP-1 that act directly in the hypothalamus to activate the sympathetic nervous system. In this minireview, we propose that leptin could be an important regulator of sympathetic innervation in reproductive tissues. Leptin may affect the density or activity of sympathetic nerves, thereby affecting reproductive function. We also speculate that other hormones such as insulin and GLP-1 may activate sympathetic nerves to the ovary. Additionally, we explore how early-onset obesity can cause lasting changes in the autonomic control of metabolic and reproductive organs, especially in the ovary. This suggests that the hyperactivation of sympathetic nerves in adulthood, due to metabolic programming, could be a possible cause of reproductive and metabolic disorders, such as polycystic ovary syndrome.

## 1 Introduction: an overview of ovarian physiology

The ovary, the organ housing female gametes, consists of ovarian follicles that serve as functional structures. Each follicle contains an oocyte surrounded by a stratified epithelium called the granulosa layer. Outside this layer lies a connective tissue layer, called the theca layer, enveloping the follicle. Both granulosa and theca cells provide nutrients and trophic support to the oocyte and produce steroid and peptide hormones that regulate various bodily functions. The process by which a follicle grows and matures is termed folliculogenesis or follicular development.

The follicle begins as an oocyte surrounded by a single layer of pregranulosa cells, forming what is known as the primordial follicle. The number of primordial follicles represents a female’s ‘follicular reserve,’ which reflects her potential reproductive capacity. In women, follicle formation occurs during gestation, while in rodents, during early postnatal life (until PND4) (1). These follicles are recruited for growth throughout life until depleted (2). The sequential transition of primordial follicles into primary, secondary, and tertiary (antral) follicles, with some selected to ovulate, is the process that determines reproductive capacity. After ovulation, the selected follicles transform into corpora lutea through the luteinization of granulosa and theca cells. All non-selected follicles are discarded via atresia, a controlled mechanism of cell death (2).

In the ovary, follicles and corpora lutea are embedded in a connective tissue rich in extracellular matrix, known as the stroma. The stroma is currently an active object of study for its diverse properties beyond just supporting the follicles. Blood vessels and nerves enter the ovary through the hilum and interweave within the stroma surrounding the follicles (3). Ovarian function, including follicle development and cyclic hormone production, relies on several internal feedback mechanisms (4), and environmental cues. In this sense, the sympathetic nervous system is a key player since it responds to various stimuli such as stress by cold exposure (5) or even the metabolic status of the body (6). In the following section, we will describe the sympathetic innervation of the ovary and provide an overview of the physiological action of this extrinsic ovarian innervation.

## 2 Sympathetic innervation of the ovary

It is well established that the ovary is innervated by autonomic nerves. The parasympathetic nerves from the Vagus nerve innervate the blood vessels. However, there is also compelling evidence of a complete and functional intracrine cholinergic system within the organ (7). In rats, sympathetic nerves reach the ovary primarily through the superior ovarian nerve (SON) and the ovarian plexus (OP) (8). The SON emerges from the suprarenal ganglion, but the SON also communicates with the ovaries through the celiac ganglion (CG), superior mesenteric ganglion (SMG), and stellate ganglion (SG), suggesting that both autonomic and sensory information from the ovaries is processed in these three ganglia (8).

Adrenergic receptors are present in the ovarian blood vessels and the follicular structures. While α-1 adrenergic receptor predominates in blood vessels, β-2 adrenergic receptors are mainly found in granulosa or luteinized cells (9). Sympathetic nerves and adrenergic receptors in the rat ovary are present from neonatal age; however, the entire functional development of these nerves occurs near puberty (10). In this sense, before puberty, sympathetic nerve release is basal and calcium-independent, while after puberty, the norepinephrine release is mostly vesicular and calcium-dependent.

Several *in vitro*, *ex vivo* and *in vivo* studies have demonstrated that adrenergic receptor pharmacological activation or blockade can modify sex hormone secretion in the ovary. For example, incubation of ovaries with the selective β-2 adrenergic agonist terbutaline increases cAMP production and induces progesterone secretion. This effect is inhibited by propranolol, a non-selective β-adrenergic antagonist, and butoxamine, a selective β-2 adrenergic antagonist, but not by practolol, a selective β-1 adrenergic antagonist (11). Additionally, isoproterenol, a non-selective β-adrenergic agonist, amplifies the response of theca-interstitial cells to hCG, thereby increasing the release of androstenedione (12).

The sympathetic nervous system (SNS) profoundly influences the regulation of follicular development. Initially, follicle development is independent of gonadotropins. Instead, neurotransmitters such as norepinephrine and vasoactive intestinal peptide (VIP) stimulate the expression of follicle-stimulating hormone receptors (FSHR) in small follicles, priming them for subsequent recruitment by FSH (13). The guanethidine-mediated sympathetic denervation alters follicle dynamics by retarding the progression of follicles, leading to abnormal estrous cycles (14). Also, the surgical denervation of the SON leads to abnormal follicle development and impaired steroid production (15). This underscores the critical role of the sympathetic nervous system in these crucial aspects of ovarian function.

In an early study conducted by Gerendai et al., using retrograde viral tracers, the authors mapped a complex neural pathway that originates in the hypothalamus, descends through the brainstem and spinal cord, and ultimately innervates the ovarian tissue via postganglionic sympathetic nerves. The study involved the injection of the pseudorabies virus into the ovary, followed by an examination of the spinal cord and brain for infected neurons. Virus-labeled nerve cells were identified using immunocytochemical techniques, revealing a polysynaptic neural route that connects the ovary to the central nervous system (CNS) and providing insights into the CNS cell groups responsible for regulating the activity of ovarian innervation (16). Interestingly, the hypothalamus, specifically the paraventricular nucleus (PVN), is one of the regions of the brain that were extensively immunolabelled, showing that PVN neurons regulate the sympathetic innervation to the ovary. The PVN is a highly integrative nucleus weighing the magnitude of different external stimuli to integrate a physiological response through the sympathetic nervous system.

## 3 Hypothalamic control of the ovarian sympathetic innervation

The PVN is a crucial region that regulates SNS activity. Often referred to as the “autonomic master controller” (17), the PVN contains two central neuronal regions: magnocellular and parvocellular. Magnocellular neurons project the posterior pituitary gland and secrete hormones such as oxytocin and vasopressin directly into the bloodstream. In contrast, parvocellular neurons can be divided into two distinct populations. One group consists of neurosecretory cells that project to the median eminence, where they release neuropeptides like corticotropin-releasing hormone (CRH) and thyrotropin-releasing hormone (TRH) into the hypothalamic-pituitary portal system. The other is the parvocellular population, which includes pre-autonomic cells that project to the medulla and spinal cord, allowing them to regulate autonomic nervous system functions (18). Some parvocellular neurons in the PVN also synthesize and release oxytocin and vasopressin (19, 20).

The PVN parvocellular neurons project to several pre-autonomic relay stations, such as the motor pressor nucleus of the rostral ventrolateral medulla (RVLM) and the Nucleus of the solitary tract (NTS). These second-order neurons in the RVLM connect with sympathetic preganglionic neurons (SPNs) in the thoracic and lumbar intermediolateral nucleus (IML) and from there to the SNS nerves in the kidneys and cardiovascular system (21, 22). Although most of the pre-autonomic neurons in the paraventricular nucleus (PVN) project to the RVLM and then to the IML (22) some of them connect directly the NTS. Mutual connections exist between the PVN and the NTS, which can control RVLM activity (23). The NTS is also involved in parasympathetic control, and evidence supports the role of PVN in controlling the RVLM by inhibiting parasympathetic effects, resulting in increased sympathetic output (24). Some spinally projecting pre-autonomic neurons (SPANs), project directly to the intermediolateral spinal columns in the IML, a central integration center and origin of motor sympathetic preganglionic neurons (SPNs) that descend ipsilaterally through the brainstem and spinal cord (21, 22, 25). The last group of pre-autonomic parvocellular neurons from the PVN innervates SPNs in the IML and sends collaterals to the RVLM, potentially having a dual role in controlling SNS output (22).

The PVN plays a role in the polysynaptic pathway that controls ovarian sympathetic innervation, which is of particular significance. This function emphasizes the broad influence of PVN on physiological processes. Specifically, thyrotropin-releasing hormone (TRH)-expressing neurons within the PVN are essential for mediating the activation of sympathetic nerves innervating the ovary (26–29). Hypothalamic manipulation of ceramides during early life in the PVN influences ovarian function without affecting GnRH control but instead influences changes in the maturation of the sympathetic nervous system in the ovary (30). This role of the PVN in controlling ovarian sympathetic innervation is a key area of research in the field of neuroendocrinology, and its implications are far-reaching.

The hypothalamic PVN receives diverse inputs from various brain regions, including the prefrontal cortex, amygdala, locus coeruleus, and hippocampus, as well as other hypothalamic nuclei such as the arcuate nucleus (ARC), ventromedial nucleus (VMN), dorsomedial nucleus (DMN), and lateral hypothalamic area (LHA) (17, 20). These inputs relay information about the bodily physiological state to the PVN. The ARC plays a central role in sensing and integrating metabolic signals related to the body’s energy status and nutrient availability (31). Two key neuron populations within the ARC detect and relay this information. POMC/CART neurons, which express pro-opiomelanocortin and cocaine- and amphetamine-regulated transcript, are activated by signals of energy sufficiency, such as elevated levels of nutrients and hormones like leptin and insulin. Activation of POMC/CART leads to the release of the neurohormone alpha-Melanocyte-stimulating hormone (α-MSH) in the PVN, promoting satiety, suppressing appetite, and increasing the activity of sympathetic nerves (32, 33). In contrast, NPY/AgRP neurons, which express neuropeptide Y and agouti-related peptide, are activated by signals of energy deficiency, such as low levels of nutrients and hormones. Activation of NPY/AgRP neurons stimulates appetite, promotes food-seeking behaviors, and decreases cardiovascular sympathetic activity and thermogenesis (34, 35). Taking together, through activating POMC/CART neurons or inhibiting NPY/AgRP neurons, leptin, insulin or other signals could activate TRH-ergic and other neurons in the PVN, which may impact the sympathetic regulation of the ovary.

## 4 Leptin control of sympathetic innervation

### 4.1 Leptin mechanism and site of action within the hypothalamus

Leptin is a hormone primarily secreted by the adipocytes of white adipose tissue that plays a crucial role in controlling food intake and energy expenditure. Leptin receptors (LepR), classified as class I cytokine receptors, including six splice variants (ObRa-f) identified to date. These receptors are expressed in various nuclei of the hypothalamus, including the ARC, VMH, LHA, and PVN, as well as extra-hypothalamic regions in the brain (36). However, leptin’s primary signaling mechanism is via the long isoform of its receptor (ObRb). Many animal models of monogenic obesity are linked to mutations in leptin or its receptors. The obese phenotype of ob/ob mice results from single mutations in leptin gene. Likewise, mutations in the leptin receptor gene explain the obese phenotypes of db/db mice, fa/fa Zucker rats, and fak/fak Koletsky rats.

Leptin reaches the brain via specific transport mediated by the LepR. In the mediobasal hypothalamus, leptin crosses the blood-cerebrospinal fluid barrier through an active transport mediated by tanycytes (37). Additionally, in other areas of the brain, leptin crosses endothelial cells lining microvessels and epithelial cells of the choroid plexus to regulate food reward rather than the homeostatic control of feeding (38).

The functions of leptin in the brain are diverse and critical. One of its key roles in maintaining energy homeostasis is the inhibition of NPY/AgRP neurons and activation of POMC/CART neurons in the ARC. POMC/CART neurons project their fibers to the PVN, where they release both α-MSH and CART. These neuropeptides not only lead to satiety but also activate the sympathetic nervous system (39, 40). This activation promotes lipolysis in white adipose tissue (WAT) (40) and thermogenesis in brown adipose tissue (BAT) (41), demonstrating the profound impact of leptin on bodily physiological processes.

### 4.2 Leptin action on the sympathetic nervous system

The catabolic function of leptin through activating the SNS is relatively predictable from a homeostatic viewpoint, as an increase in leptin due to fat accumulation leads the organism to be more prone to thermogenesis and fat catabolism (41–43). However, intracerebroventricular (ICV) leptin injection also increases sympathetic nerve activity in other organs such as kidneys, thus increasing blood pressure (44). In addition, ICV leptin injection increases norepinephrine levels in the liver and the ovary (45). In this sense, leptin control of sympathetic activity goes beyond its role in energy homeostasis, acting as a periphery-brain signal for other physiological functions.

Different nuclei of the hypothalamus have different influences on sympathetic innervation and several studies have shown this difference depending on the nuclei in which leptin was microinjected. Leptin microinjection into the ARC, but not into VMH, increased hepatic sympathetic activity conveying specifically in phosphatidylinositol 3-kinase (PI3K) but not on AMP-activated protein kinase (AMPK), STAT3, or ERK1/2 pathways (46). Interestingly, the blockade of PI3K prevents leptin-induced sympathetic activation to the kidney but not in BAT, lumbar, or adrenal glands (47). Furthermore, ICV administration of leptin activates hepatic AMPK through sympathetic nerves, but hepatic vagotomy does not affect this activation (48). In contrast, chemical sympathectomy inhibited α1-adrenergic receptors-induced AMPK activation in the liver induced by leptin (48), differing on the lipolytic effects of leptin in WAT, where β-adrenergic receptors are required (42). On the other side, the blockade of ERK1/2 eliminates leptin-induced increases in sympathetic nerve activity in the BAT. However, it does not alter the stimulatory effects of leptin on sympathetic nerve activity in the kidney, lumbar, or adrenal gland (47). Another study showed that microinjection of leptin into the commissural and medial subnuclei of the caudal NTS increased renal but not BAT sympathetic nerve activity (49).

We have recently shown that sub-chronic ICV leptin injection increases norepinephrine levels in the ovary (45). Since TRH release from the magnocellular PVN induces the activation of the SNS nerves arriving into the ovary (29), our results suggest that leptin-mediated activation of TRH neurons in the PVN could be the source of the leptin-induced release of norepinephrine in the ovary. However, it remains unclear whether leptin directly activates the TRH-ergic neurons, or if it does it indirectly by activating POMC neurons in the ARH, or possibly both. The latter possibility is plausible, as both hypophysiotropic and pre-autonomic TRH-ergic neurons of the PVN are innervated by POMC/CART and NPY/AgRP neurons of the ARC (50). Interestingly, the knockdown of LepR in this area does not decrease the tonic regulation of cardiovascular function via the sympathetic nervous system, but it does stimulate food intake (51). Finally, obesity may alter the PVN-SNS-ovary pathway through increasing leptin. Indeed, we observed that early-onset obesity in rats increases NE levels in the ovary when they are adults, which occurs alongside high leptin levels (45). This same pathway may be involved in the precocious puberty induced by obesity (30).

**Figure 1:**
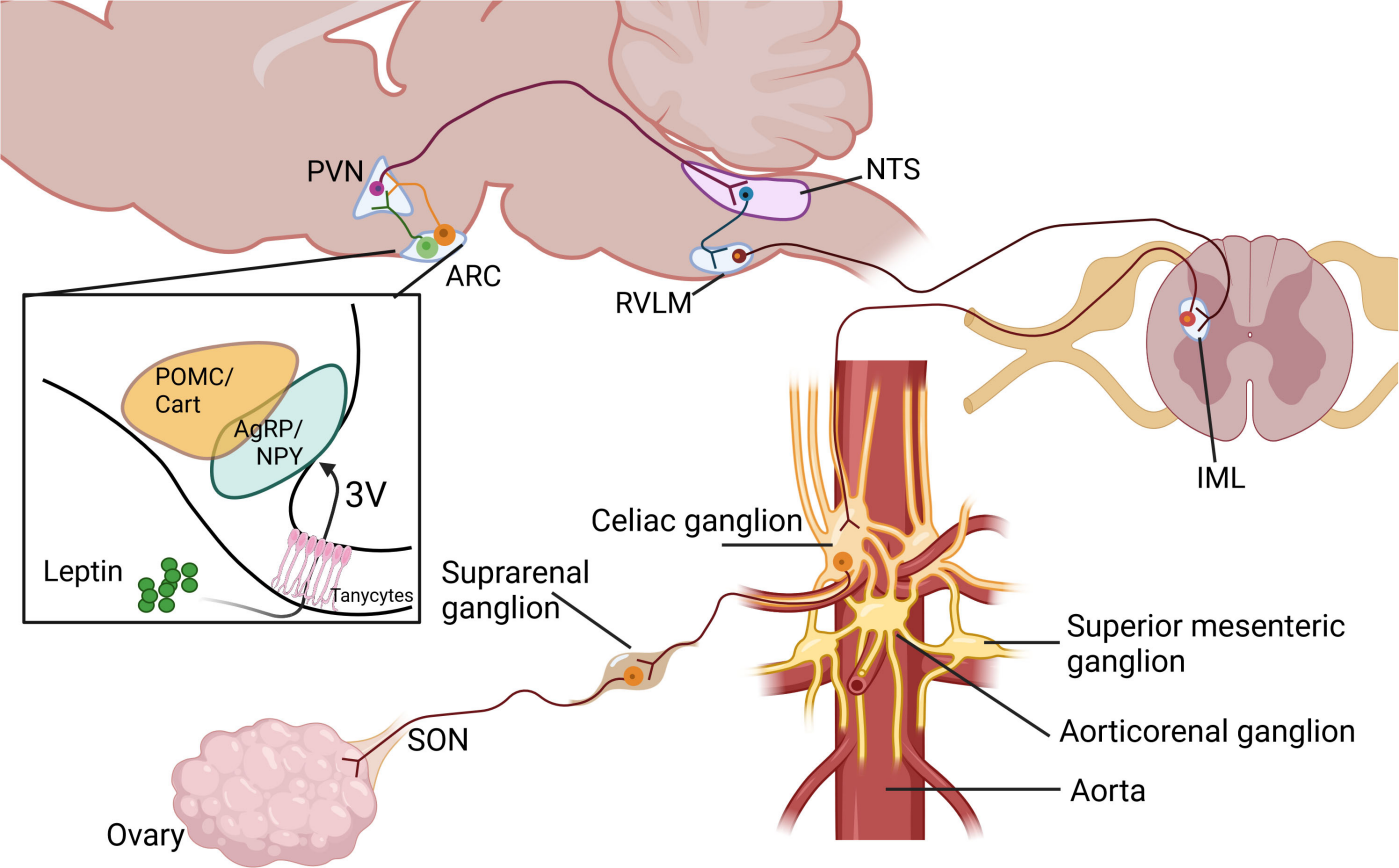
Proposed pathways in the control of ovarian function by leptin through the sympathetic nervous system. Paraventricular nucleus of the hypothalamus (PVN), Arcuate nucleus of the hypothalamus (ARC), Nucleus of the solitary tract (NTS), Rostral ventrolateral medulla (RVLM), Intermediolateral cell column at spinal cord (IML), Superior Ovarian Nerve (SON). Created in BioRender. Fernandois, D. (2025) https://BioRender.com/x73b564.

Collectively, these studies underscore the intricate and multifaceted nature of brain leptin action and its diverse physiological effects. Leptin triggers different signaling pathways at the hypothalamus and the leptin-dependent SNS activation in different tissues depends on different hypothalamic nuclei. **Figure 1** illustrates a proposed model of how Leptin, through its action on the brain, controls ovarian function.

### 4.3 Developmental reprogramming of sympathetic innervation by maternal obesity: role of leptin

Maternal obesity during pregnancy and lactation can affect hormone secretion, metabolite concentrations, and the availability of nutrients to the fetus or infant. Consequently, these hormones, metabolites, and nutrients can impact the development of the offspring’s organs. We and other groups have shown that the offspring of obese mothers have elevated plasmatic levels of leptin during infancy (52, 53). Interestingly, leptin is not effective in reducing milk consumption during infancy in rats (54), but it does impact metabolic rate. Notably, leptin functions as a neurotrophic factor during brain neurodevelopment, rather than solely regulating energy balance (55, 56). Since hypothalamic circuits controlling energy balance mature during infancy (57), increased levels of neurotrophic factors such as estradiol or leptin during this critical period can negatively impact the development of hypothalamic circuits controlling energy balance and the hypothalamic connections to the SNS. In this context, previous research has shown that the offspring of rats fed a high-fat diet during pregnancy and lactation, which models maternal obesity, exhibit increased estradiol (58) and leptin levels (52, 53) during early development. This model also exhibits increased norepinephrine levels in the ovary (52) and kidneys (59) in the offspring. Therefore, leptin levels during postnatal development may be critical for correctly establishing hypothalamic regulation of the sympathetic outflow to the organs. In other words, the offspring of obese mothers may have an elevated set-point regulation for the sympathetic tone and increased innervation of these organs due to increased leptin levels during infancy. That leads to a higher susceptibility to developing chronic diseases when exposed to environmental challenges (i.e., stress, overfeeding) during adulthood. Indeed, offspring of obese mothers have increased risk factors for developing hypertension (59), polycystic ovaries (52, 58), and non-alcoholic fatty liver disease (60), all pathologies that are associated with hyperactivation of the SNS.

## 5 A potential role of hormones beyond leptin in hypothalamic modulation of sympathetic pathways to the ovary

### 5.1 Insulin

Insulin is released from the pancreas in response to elevated blood glucose levels and primarily acts on the liver, muscles, and adipose tissue to regulate blood glucose levels in a homeostatic manner. Insulin also reaches the hypothalamus, where it induces satiety and influences specific nuclei involved in the sympathetic pathways to the ovary. Importantly, insulin activates at least two excitatory inputs into the paraventricular nucleus (PVN). The first is α-melanocyte-stimulating hormone (α-MSH), which binds to melanocortin type 3 and 4 receptors (MC3/4R) (61). The second is glutamate, which binds to NMDA receptors (62). Additionally, insulin inhibits the primary inhibitory input to the PVN, which comes from neuropeptide Y (NPY) projections originating in the arcuate nucleus (ARC) (32). Both the action of leptin and insulin are even potentiated by another hormone, Angiotensin II, to increase the excitation of sympathetic fibers (63). Interestingly, despite insulin resistance and decreased transport of insulin across the blood-brain barrier, the brain becomes more sensitive to the sympathoexcitatory effects and pressure action of insulin in obesity, through an unknown mechanism (64). It is still unclear whether the insulin action on ARC-PVN-SNS pathway controls ovarian physiology. Future research should clarify whether brain insulin action influences ovarian physiology and contributes to ovarian pathology through the sympathetic nervous system.

### 5.2 Glucagon like peptide 1

Glucagon-like peptide 1 (GLP-1) is an incretin secreted from the gut after a meal. It increases insulin secretion from the pancreas and has wide effects on the body. Additionally, neurons that release GLP-1 are found in the nucleus of the solitary tract (NTS), ventrolateral medulla, and olfactory bulb (65, 66). These neurons project to the hypothalamus and other brain regions (67, 68). GLP-1 receptors are highly expressed in the PVN and arcuate nucleus (69, 70), both nuclei involved in controlling pre-autonomic neurons in the brain. Indeed, GLP-1agonists injections in the PVN lead to increase renal sympathetic activation and mean arterial pressure demonstrating an activation on sympathetic nerves (70). Alternatively, the action of GLP-1 on the olfactory bulb inhibits PVN neurons lead to decrease sympathetic tone in the pancreas (71). Regarding the ovary, several reports have proposed GLP-1 agonists to treat PCOS, which is still on research. Certainly, GLP-1 has demonstrated direct and indirect effects on the ovary. In example, GLP-1 administration to rats increases follicular atresia and alters the redox balance of the ovary (72). Also, GLP-1 or exendin-4, a GLP-1R agonist, modulates hypothalamus-pituitary gonad axis, modifying follicle development (73). On the other hand, GLP-1-based multi-agonists have demonstrated notable beneficial effects. Specifically, the GLP-1/Estrogen combination has shown superior efficacy compared to metformin and other multi-agonists in managing the metabolic complications of PCOS, while also enhancing ovarian cyclicity in an anovulatory PCOS model (74). Although hypothalamic GLP-1 regulates sympathetic innervation to various organs, the effect of GLP-1 on regulating sympathetic innervation of the ovary remains speculative and needs to be demonstrated.

## 6 Role of the PVN- SNS-ovary pathway in ovarian pathophysiology and treatment

It is well known that polycystic ovary syndrome (PCOS) in humans is associated with increased sympathetic nervous system activity and a heightened cardiovascular risk. In animal models of PCOS, which can be induced by early administration of androgens or estrogens, an increase in the activity of ovarian sympathetic nerves has been observed (75, 76). Notably, denervation of the sympathetic nerves to the ovaries can partially restore ovulation, improve estrous cyclicity, and enhance follicle development (77). Additionally, insulin resistance and leptin resistance, along with hyperleptinemia and hyperinsulinemia, are also present in animal models of PCOS (74, 78, 79). Consequently, elevated levels of leptin and compensatory hyperinsulinemia may lead to increased activity in the sympathetic nerves that innervate the ovaries. Moreover, interventions such as weight loss, regular physical exercise, metformin, and GLP-1 agonists have demonstrated favorable outcomes in enhancing follicle development, promoting ovulation, and alleviating the symptoms associated with PCOS (80–84). All of these treatments improve insulin sensitivity, leading to decreased insulin and leptin levels due to a reduction in body fat. However, it remains uncertain whether these improvements also reduce sympathetic activity in the ovaries or other organs. In summary, these insights emphasize the need for further research to determine whether the modulation of sympathetic nerve activity through lowered leptin or insulin levels plays a central role in the effects of these treatments.

## 7 Concluding remarks

In the complex relationship between metabolism and reproduction, leptin plays a crucial role in regulating how metabolism affects reproduction by controlling the endocrine HPG axis. Our proposal suggests that leptin also influences the sympathetic innervation of the ovary, leading to increased neural activity. This implies that in cases of obesity, leptin’s altered control of the SNS could significantly impact ovarian function and contribute to conditions like PCOS. Additionally, early exposure to obesity or overfeeding may raise leptin levels during early development, potentially changing the relationship between the hypothalamus and SNS. This could result in an increased susceptibility to chronic diseases in offspring due to overactivation of the SNS.
